# (1*S*,2*R*,7*R*,8*S*,10*R*)-9,9-Di­bromo-2,6,6,10-tetra­methyl-1α,2α-ep­oxy­tri­cyclo­[5.5.0.0^8,10^]dodeca­ne

**DOI:** 10.1107/S160053681301502X

**Published:** 2013-06-08

**Authors:** Ahmed Benharref, Jamal El Karroumi, Lahcen El Ammari, Mohamed Saadi, Moha Berraho

**Affiliations:** a Laboratoire de Chimie des Substances Naturelles, "Unité Associé au CNRST (URAC16)", Faculté des Sciences Semlalia, BP 2390 Bd My Abdellah, 40000 Marrakech, Morocco; b Laboratoire de Chimie du Solide, Appliquée, Faculté des Sciences, Université Mohammed V-Agdal , Avenue Ibn Battouta, BP 1014, Rabat, Morocco

## Abstract

The title compound, C_16_H_24_Br_2_O, was synthesized from the reaction of β-himachalene (3,5,5,9-tetra­methyl-2,4a,5,6,7,8-hexa­hydro-1*H*-benzo­cyclo­heptene), which was isolated from Atlas cedar (*Cedrus atlantica*) essential oil, after reaction with di­bromo­carbene. The asymmetric unit contains two independent mol­ecules with similar conformations. Each mol­ecule is built up from fused six-and seven-membered rings and two three-membered rings. In both mol­ecules, the six-membered ring has an envelope conformation with the flap provided by the C atom of the ep­oxy ring, whereas the seven-membered ring displays a chair conformation. The crystal packing is governed only by van der Waals inter­actions. The absolute configuration was established from anomalous dispersion effects.

## Related literature

For background to β-himachalene, see: Benharref *et al.*(2013 ▶); Oukhrib *et al.*(2013*a* ▶,*b* ▶). For the reactivity of this sesquiterpene and its derivatives, see: El Haib *et al.* (2011 ▶). For details of the synthesis, see: El Jamili *et al.* (2002 ▶). For puckering parameters, see: Cremer & Pople (1975 ▶).
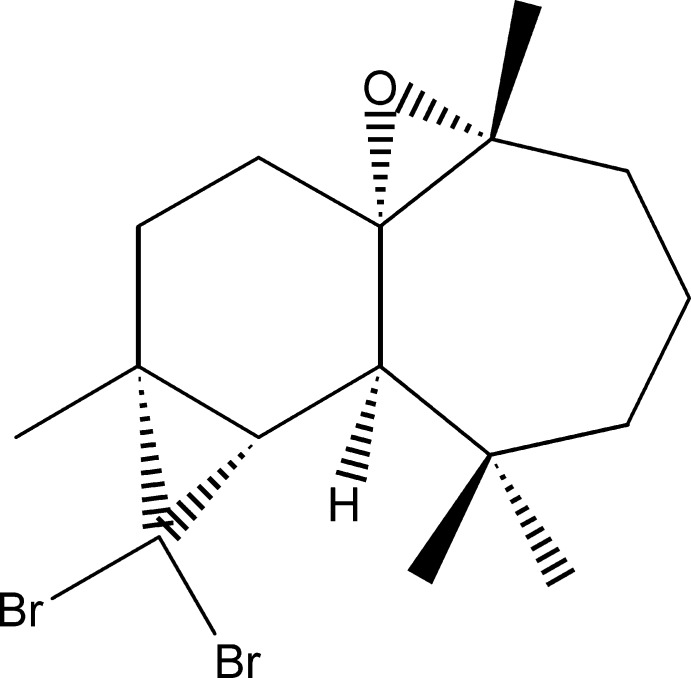


## Experimental

### 

#### Crystal data

C_16_H_24_Br_2_O
*M* *_r_* = 392.17
Monoclinic, 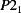

*a* = 8.8056 (13) Å
*b* = 15.648 (3) Å
*c* = 12.1390 (16) Å
β = 91.769 (10)°
*V* = 1671.8 (4) Å^3^
*Z* = 4
Mo *K*α radiation
μ = 4.84 mm^−1^
*T* = 293 K
0.25 × 0.15 × 0.10 mm

#### Data collection

Bruker APEXII CCD diffractometer
Absorption correction: multi-scan (*SADABS*; Bruker, 2009 ▶) *T*_min_ = 0.423, *T*_max_ = 0.617
17250 measured reflections
6776 independent reflections
5298 reflections with *I* > 2σ(*I*)
*R*_int_ = 0.081

#### Refinement

*R*[*F* ^2^ > 2σ(*F* ^2^)] = 0.045
*wR*(*F* ^2^) = 0.108
*S* = 0.96
6776 reflections
351 parameters
1 restraint
H-atom parameters constrained
Δρ_max_ = 0.71 e Å^−3^
Δρ_min_ = −0.67 e Å^−3^
Absolute structure: Flack & Bernardinelli (2000 ▶)
Flack parameter: 0.009 (10)

### 

Data collection: *APEX2* (Bruker, 2009 ▶); cell refinement: *SAINT* (Bruker, 2009 ▶); data reduction: *SAINT*; program(s) used to solve structure: *SHELXS97* (Sheldrick,2008 ▶); program(s) used to refine structure: *SHELXL97* (Sheldrick,2008 ▶); molecular graphics: *ORTEP-3 for Windows* (Farrugia, 2012 ▶); software used to prepare material for publication: *WinGX* publication routines (Farrugia, 2012 ▶).

## Supplementary Material

Crystal structure: contains datablock(s) global. DOI: 10.1107/S160053681301502X/rz5070sup1.cif

Structure factors: contains datablock(s) I. DOI: 10.1107/S160053681301502X/rz5070Isup2.hkl

Supplementary material file. DOI: 10.1107/S160053681301502X/rz5070Isup3.cml

Additional supplementary materials:  crystallographic information; 3D view; checkCIF report

## Figures and Tables

**Fig. 1.**
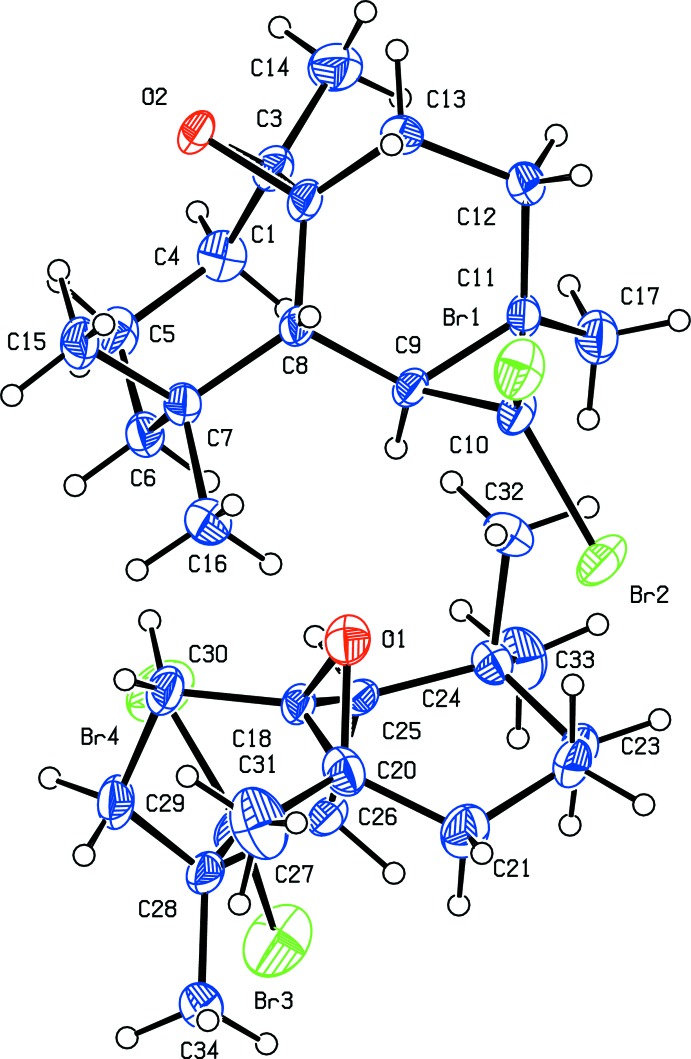
The molecular structure of the title compound with displacement ellipsoids drawn at the 30% probability level. H atoms are represented as small spheres of arbitrary radii.

## supplementary crystallographic information

### Comment

As part of the development of the essential oil of Atlas cedar (*Cedrus atlantica*) made up mainly (50%) of β-himachalene (Benharref *et al.*, 2013; Oukhrib *et al.*, 2013*a*,*b*). The reactivity of this sesquiterpene and its derivatives has been studied extensively by our team in order to prepare new products having biological proprieties (El Haib *et al.*, 2011). We present in this paper the crystal structure of the title compound (1*S*,2*R*,7*R* 8S,10*R*)-9,9- dibromo-1α,2α-epoxy-2,6,6,10-tetramethyltricyclo[5.5.0.0^8^,^10^]dodecane. The asymmetric unit of the title compound contains two independent molecules of similar geometry (Fig. 1). Each molecule contains a fused six- and a seven-membered ring, which are fused to two three-membered rings as shown in Fig. 1. The six-membered ring has an envelope conformation, as indicated by the total puckering amplitude QT = 0.622 (6) Å and spherical polar angle θ = 120.37 (5)° with φ = 176.92 (6)°, whereas the seven-membered ring displays a chair conformation with QT = 0.626 (5) Å, θ = 22.71 (5)°, φ2 = 149.10 (14)° and φ3 = 102.01 (6)° (Cremer & Pople, 1975). Owing to the presence of Br atoms, the absolute configuration could be fully confirmed, by refining the Flack parameter (Flack & Bernardinelli, 2000) as C1(*S*), C2(*R*), C7(*R*), 8(*S*) and 10(*R*).

### Experimental

A solution containing 2 g (9 mmol) of 6α,7α-epoxyhimachalene ((1*S*,2*R*,7*R*)-2,6,6,9-tetramethylbicyclo[5.4.0.]dec-8-ene) (El Jamili *et al.*, 2002) and 1 ml (10 mmol) of CHBr_3_ in 40 ml of dichloromethane was added dropwise at 273 K over 30 min to 1 g of pulverized sodium hydroxide and 40 mg of *N*-benzyltriethylammonium chloride placed in a 100 ml three-necked flask. After stirring at room temperature for 2 h, the mixture was filtered on celite and concentrated in vacuum. The residue obtained was chromatographed on silica gel column impregnated with silver nitrate (10%) with a mixture of hexane-ethyl acetate (95:5 *v*/*v*) used as eluent. The two diastereoisomers (1*S*,2*R*,7*R*,8*S*,10*R*)-9,9-dibromo- 1α,2α-epoxy-2,6,6,10-tetramethyltricyclo[5.5.0.0^8^,^10^]dodecane (*X*) and its isomer (1*R*,2*R*,7*R*,8*R*,10*S*)-9,9-dibromo-1α,2α-epoxy-2,6,6,10-tetramethyltricyclo[5.5.0.0^8^,^10^]dodecane (*Y*), were obtained by this procedure in a 80/20 ratio and a combined yield of 85% (3 g; 7.6 mmol). The title compound (isomer *X*) was recrystallized from heptane.

### Refinement

All H atoms were fixed geometrically and treated as riding with C—H = 0.96 Å (methyl), 0.97 Å (methylene), 0.98 Å (methine) with *U*_iso_(H) = 1.2*U*_eq_(C) or *U*_iso_(H) = 1.5*U*_eq_(C) for methyl H atoms.

### Crystal data

**Table:**

C_16_H_24_Br_2_O	*F*(000) = 792
*M**_r_* = 392.17	*D*_x_ = 1.558 Mg m^−^^3^
Monoclinic, *P*2_1_	Mo *K*α radiation, λ = 0.71073 Å
Hall symbol: P 2yb	Cell parameters from 6776 reflections
*a* = 8.8056 (13) Å	θ = 1.7–26.4°
*b* = 15.648 (3) Å	µ = 4.84 mm^−^^1^
*c* = 12.1390 (16) Å	*T* = 293 K
β = 91.769 (10)°	Block, colourless
*V* = 1671.8 (4) Å^3^	0.25 × 0.15 × 0.10 mm
*Z* = 4	

### Data collection

**Table:**

Bruker APEXII CCD diffractometer	6776 independent reflections
Radiation source: fine-focus sealed tube	5298 reflections with *I* > 2σ(*I*)
Graphite monochromator	*R*_int_ = 0.081
ω and φ scans	θ_max_ = 26.4°, θ_min_ = 1.7°
Absorption correction: multi-scan (*SADABS*; Bruker, 2009)	*h* = −8→11
*T*_min_ = 0.423, *T*_max_ = 0.617	*k* = −19→19
17250 measured reflections	*l* = −15→15

### Refinement

**Table:**

Refinement on *F*^2^	Secondary atom site location: difference Fourier map
Least-squares matrix: full	Hydrogen site location: inferred from neighbouring sites
*R*[*F*^2^ > 2σ(*F*^2^)] = 0.045	H-atom parameters constrained
*wR*(*F*^2^) = 0.108	*w* = 1/[σ^2^(*F*_o_^2^) + (0.0633*P*)^2^] where *P* = (*F*_o_^2^ + 2*F*_c_^2^)/3
*S* = 0.96	(Δ/σ)_max_ = 0.001
6776 reflections	Δρ_max_ = 0.71 e Å^−^^3^
351 parameters	Δρ_min_ = −0.67 e Å^−^^3^
1 restraint	Absolute structure: Flack & Bernardinelli (2000)
Primary atom site location: structure-invariant direct methods	Flack parameter: 0.009 (10)

### Special details

**Table:**

Geometry. All s.u.'s (except the s.u. in the dihedral angle between two l.s. planes) are estimated using the full covariance matrix. The cell s.u.'s are taken into account individually in the estimation of s.u.'s in distances, angles and torsion angles; correlations between s.u.'s in cell parameters are only used when they are defined by crystal symmetry. An approximate (isotropic) treatment of cell s.u.'s is used for estimating s.u.'s involving l.s. planes.
Refinement. Refinement of *F*^2^ against all reflections. The weighted *R*-factor *wR* and goodness of fit *S* are based on *F*^2^, conventional *R*-factors *R* are based on *F*, with *F* set to zero for negative *F*^2^. The threshold expression of *F*^2^ > 2σ(*F*^2^) is used only for calculating *R*-factors(gt) *etc*. and is not relevant to the choice of reflections for refinement. *R*-factors based on *F*^2^ are statistically about twice as large as those based on *F*, and *R*- factors based on all data will be even larger.

### Fractional atomic coordinates and isotropic or equivalent isotropic displacement parameters (Å^2^)

**Table:**

	*x*	*y*	*z*	*U*_iso_*/*U*_eq_	
C1	0.4786 (5)	0.2569 (3)	0.7030 (3)	0.0390 (10)	
O2	0.5953 (4)	0.2243 (2)	0.7806 (2)	0.0430 (8)	
C2	0.5383 (6)	0.1681 (3)	0.6920 (4)	0.0423 (11)	
C3	0.6486 (6)	0.1431 (3)	0.6069 (4)	0.0487 (12)	
H3A	0.5939	0.1412	0.5363	0.058*	
H3B	0.6823	0.0853	0.6233	0.058*	
C4	0.7899 (6)	0.1980 (3)	0.5929 (4)	0.0488 (12)	
H4A	0.8350	0.2091	0.6654	0.059*	
H4B	0.8628	0.1651	0.5522	0.059*	
C5	0.7662 (5)	0.2824 (3)	0.5350 (4)	0.0444 (11)	
H5A	0.7130	0.2712	0.4653	0.053*	
H5B	0.8654	0.3049	0.5180	0.053*	
C6	0.6796 (5)	0.3524 (3)	0.5942 (3)	0.0388 (10)	
C7	0.5144 (5)	0.3291 (3)	0.6248 (3)	0.0328 (9)	
H7	0.4749	0.3806	0.6599	0.039*	
C8	0.4079 (5)	0.3138 (3)	0.5248 (3)	0.0325 (9)	
H8	0.4592	0.2942	0.4589	0.039*	
C9	0.2679 (5)	0.3648 (3)	0.5012 (3)	0.0401 (11)	
C10	0.2532 (5)	0.2739 (3)	0.5400 (4)	0.0372 (10)	
C11	0.1999 (5)	0.2574 (4)	0.6582 (4)	0.0473 (12)	
H11A	0.1646	0.1988	0.6631	0.057*	
H11B	0.1145	0.2945	0.6722	0.057*	
C12	0.3252 (5)	0.2725 (4)	0.7486 (4)	0.0448 (11)	
H12A	0.3193	0.3308	0.7754	0.054*	
H12B	0.3096	0.2343	0.8101	0.054*	
C13	0.4477 (8)	0.0924 (4)	0.7295 (5)	0.0633 (16)	
H13A	0.5153	0.0501	0.7604	0.095*	
H13B	0.3918	0.0686	0.6677	0.095*	
H13C	0.3782	0.1105	0.7843	0.095*	
C14	0.7687 (6)	0.3802 (4)	0.6983 (4)	0.0490 (12)	
H14A	0.8660	0.4021	0.6785	0.074*	
H14B	0.7828	0.3320	0.7465	0.074*	
H14C	0.7132	0.4240	0.7353	0.074*	
C15	0.6689 (6)	0.4301 (3)	0.5177 (4)	0.0536 (13)	
H15A	0.6141	0.4750	0.5529	0.080*	
H15B	0.6166	0.4143	0.4502	0.080*	
H15C	0.7693	0.4496	0.5020	0.080*	
C16	0.1993 (6)	0.2064 (4)	0.4603 (4)	0.0546 (13)	
H16A	0.0903	0.2074	0.4543	0.082*	
H16B	0.2326	0.1514	0.4861	0.082*	
H16C	0.2403	0.2173	0.3893	0.082*	
C17	0.2827 (5)	0.7233 (3)	0.8031 (3)	0.0352 (10)	
C18	0.3421 (6)	0.8076 (3)	0.8327 (4)	0.0465 (12)	
C19	0.4705 (7)	0.8218 (3)	0.9162 (5)	0.0561 (14)	
H19A	0.4288	0.8165	0.9890	0.067*	
H19B	0.5044	0.8804	0.9087	0.067*	
C20	0.6092 (6)	0.7649 (4)	0.9129 (4)	0.0497 (13)	
H20A	0.6433	0.7636	0.8377	0.060*	
H20B	0.6895	0.7911	0.9579	0.060*	
C21	0.5900 (6)	0.6742 (3)	0.9511 (4)	0.0459 (12)	
H21A	0.5452	0.6760	1.0231	0.055*	
H21B	0.6905	0.6495	0.9611	0.055*	
C22	0.4959 (5)	0.6139 (3)	0.8797 (4)	0.0398 (10)	
C23	0.3285 (5)	0.6408 (3)	0.8609 (3)	0.0323 (9)	
H23	0.2826	0.5952	0.8156	0.039*	
C24	0.2385 (5)	0.6414 (3)	0.9667 (3)	0.0368 (10)	
H24	0.2997	0.6538	1.0336	0.044*	
C25	0.1056 (7)	0.5875 (4)	0.9849 (4)	0.0575 (15)	
C26	0.0784 (5)	0.6799 (4)	0.9657 (4)	0.0464 (12)	
C27	0.0114 (6)	0.7110 (5)	0.8544 (4)	0.0603 (15)	
H27A	−0.0736	0.6743	0.8337	0.072*	
H27B	−0.0285	0.7681	0.8644	0.072*	
C28	0.1207 (5)	0.7130 (4)	0.7588 (4)	0.0498 (12)	
H28A	0.1117	0.6603	0.7169	0.060*	
H28B	0.0947	0.7602	0.7100	0.060*	
C29	0.2462 (8)	0.8869 (4)	0.8139 (6)	0.0794 (19)	
H29A	0.3107	0.9341	0.7963	0.119*	
H29B	0.1925	0.8997	0.8796	0.119*	
H29C	0.1746	0.8771	0.7540	0.119*	
C30	0.5677 (7)	0.6043 (4)	0.7651 (4)	0.0562 (14)	
H30A	0.5677	0.6588	0.7286	0.084*	
H30B	0.5096	0.5642	0.7214	0.084*	
H30C	0.6703	0.5842	0.7744	0.084*	
C31	0.4967 (8)	0.5255 (4)	0.9344 (5)	0.0686 (16)	
H31A	0.5984	0.5034	0.9374	0.103*	
H31B	0.4321	0.4875	0.8923	0.103*	
H31C	0.4600	0.5304	1.0078	0.103*	
C32	0.0383 (7)	0.7370 (5)	1.0609 (5)	0.0702 (18)	
H32A	−0.0685	0.7327	1.0733	0.105*	
H32B	0.0635	0.7951	1.0437	0.105*	
H32C	0.0945	0.7194	1.1260	0.105*	
Br1	0.21034 (6)	0.45774 (4)	0.59468 (5)	0.05955 (16)	
Br2	0.21696 (7)	0.39320 (4)	0.34957 (4)	0.06233 (17)	
Br3	0.08341 (10)	0.54023 (6)	1.13196 (5)	0.0965 (3)	
Br4	0.03361 (10)	0.50475 (5)	0.87765 (6)	0.0890 (3)	
O1	0.3873 (4)	0.7654 (2)	0.7306 (2)	0.0488 (8)	

### Atomic displacement parameters (Å^2^)

**Table:**

	*U*^11^	*U*^22^	*U*^33^	*U*^12^	*U*^13^	*U*^23^
C1	0.031 (2)	0.058 (3)	0.028 (2)	0.000 (2)	−0.0064 (18)	−0.0019 (19)
O2	0.0350 (18)	0.062 (2)	0.0313 (15)	0.0037 (15)	−0.0106 (13)	0.0048 (14)
C2	0.043 (3)	0.045 (3)	0.038 (2)	−0.004 (2)	−0.008 (2)	−0.001 (2)
C3	0.052 (3)	0.040 (3)	0.054 (3)	0.005 (2)	0.001 (2)	−0.004 (2)
C4	0.037 (3)	0.060 (3)	0.050 (3)	0.014 (2)	0.003 (2)	−0.007 (2)
C5	0.030 (2)	0.066 (3)	0.037 (2)	−0.002 (2)	0.002 (2)	−0.003 (2)
C6	0.031 (2)	0.051 (3)	0.034 (2)	−0.003 (2)	−0.0013 (18)	−0.0012 (19)
C7	0.028 (2)	0.047 (3)	0.0230 (19)	0.0014 (19)	−0.0013 (16)	−0.0066 (17)
C8	0.030 (2)	0.043 (2)	0.0243 (19)	0.0032 (18)	−0.0046 (17)	−0.0004 (17)
C9	0.035 (2)	0.053 (3)	0.032 (2)	0.010 (2)	−0.0056 (18)	−0.0069 (19)
C10	0.025 (2)	0.052 (3)	0.035 (2)	0.000 (2)	−0.0035 (18)	−0.003 (2)
C11	0.030 (2)	0.073 (3)	0.039 (2)	−0.003 (2)	0.002 (2)	0.005 (2)
C12	0.036 (3)	0.067 (3)	0.032 (2)	0.003 (2)	0.007 (2)	0.006 (2)
C13	0.069 (4)	0.063 (4)	0.057 (3)	−0.013 (3)	−0.004 (3)	0.014 (3)
C14	0.036 (3)	0.061 (3)	0.049 (3)	−0.008 (2)	−0.009 (2)	−0.006 (2)
C15	0.048 (3)	0.056 (3)	0.057 (3)	−0.008 (2)	0.000 (2)	0.011 (2)
C16	0.042 (3)	0.069 (3)	0.053 (3)	−0.004 (3)	−0.004 (2)	−0.007 (3)
C17	0.027 (2)	0.054 (3)	0.0245 (19)	−0.002 (2)	−0.0018 (16)	0.0048 (18)
C18	0.038 (3)	0.051 (3)	0.050 (3)	0.009 (2)	0.003 (2)	0.007 (2)
C19	0.054 (3)	0.046 (3)	0.068 (3)	−0.008 (3)	−0.009 (3)	−0.010 (2)
C20	0.034 (3)	0.067 (3)	0.048 (3)	−0.009 (2)	−0.008 (2)	−0.010 (2)
C21	0.036 (3)	0.065 (3)	0.037 (2)	0.004 (2)	−0.007 (2)	0.004 (2)
C22	0.035 (3)	0.041 (3)	0.043 (2)	0.005 (2)	0.003 (2)	0.0016 (19)
C23	0.032 (2)	0.038 (2)	0.027 (2)	−0.0050 (18)	−0.0015 (17)	−0.0048 (17)
C24	0.036 (2)	0.049 (3)	0.0256 (19)	−0.011 (2)	−0.0029 (18)	−0.0008 (18)
C25	0.059 (4)	0.077 (4)	0.037 (2)	−0.037 (3)	0.000 (2)	0.002 (2)
C26	0.028 (3)	0.080 (4)	0.032 (2)	−0.013 (2)	0.0011 (18)	−0.007 (2)
C27	0.027 (3)	0.104 (5)	0.049 (3)	−0.008 (3)	−0.004 (2)	0.001 (3)
C28	0.036 (3)	0.078 (4)	0.034 (2)	0.001 (3)	−0.009 (2)	0.005 (2)
C29	0.072 (4)	0.050 (3)	0.116 (5)	0.016 (3)	0.003 (4)	0.019 (4)
C30	0.049 (3)	0.066 (4)	0.054 (3)	0.013 (3)	0.011 (3)	−0.009 (3)
C31	0.065 (4)	0.054 (4)	0.086 (4)	0.014 (3)	−0.002 (3)	0.010 (3)
C32	0.037 (3)	0.121 (6)	0.052 (3)	0.005 (3)	0.004 (2)	−0.021 (3)
Br1	0.0545 (3)	0.0613 (3)	0.0625 (3)	0.0171 (3)	−0.0040 (2)	−0.0144 (3)
Br2	0.0657 (4)	0.0773 (4)	0.0426 (3)	0.0070 (3)	−0.0198 (2)	0.0090 (3)
Br3	0.0981 (6)	0.1335 (7)	0.0583 (4)	−0.0518 (5)	0.0060 (4)	0.0353 (4)
Br4	0.0915 (5)	0.0958 (5)	0.0797 (4)	−0.0565 (4)	0.0018 (4)	−0.0205 (4)
O1	0.044 (2)	0.068 (2)	0.0352 (16)	−0.0027 (17)	0.0022 (15)	0.0158 (15)

### Geometric parameters (Å, º)

**Table:**

C1—O2	1.465 (5)	C17—O1	1.450 (5)
C1—C2	1.493 (7)	C17—C18	1.459 (7)
C1—C12	1.495 (7)	C17—C28	1.517 (6)
C1—C7	1.515 (7)	C17—C23	1.519 (6)
O2—C2	1.466 (5)	C18—O1	1.470 (6)
C2—C3	1.492 (7)	C18—C19	1.512 (7)
C2—C13	1.506 (7)	C18—C29	1.514 (8)
C3—C4	1.526 (8)	C19—C20	1.514 (8)
C3—H3A	0.9700	C19—H19A	0.9700
C3—H3B	0.9700	C19—H19B	0.9700
C4—C5	1.507 (8)	C20—C21	1.504 (8)
C4—H4A	0.9700	C20—H20A	0.9700
C4—H4B	0.9700	C20—H20B	0.9700
C5—C6	1.528 (7)	C21—C22	1.511 (7)
C5—H5A	0.9700	C21—H21A	0.9700
C5—H5B	0.9700	C21—H21B	0.9700
C6—C14	1.530 (6)	C22—C31	1.535 (8)
C6—C15	1.530 (7)	C22—C23	1.543 (6)
C6—C7	1.556 (6)	C22—C30	1.554 (7)
C7—C8	1.530 (5)	C23—C24	1.530 (6)
C7—H7	0.9800	C23—H23	0.9800
C8—C9	1.489 (6)	C24—C25	1.464 (7)
C8—C10	1.514 (6)	C24—C26	1.532 (7)
C8—H8	0.9800	C24—H24	0.9800
C9—C10	1.504 (7)	C25—C26	1.482 (9)
C9—Br1	1.922 (4)	C25—Br4	1.930 (5)
C9—Br2	1.933 (4)	C25—Br3	1.948 (5)
C10—C16	1.500 (7)	C26—C32	1.510 (7)
C10—C11	1.545 (6)	C26—C27	1.536 (7)
C11—C12	1.551 (7)	C27—C28	1.531 (7)
C11—H11A	0.9700	C27—H27A	0.9700
C11—H11B	0.9700	C27—H27B	0.9700
C12—H12A	0.9700	C28—H28A	0.9700
C12—H12B	0.9700	C28—H28B	0.9700
C13—H13A	0.9600	C29—H29A	0.9600
C13—H13B	0.9600	C29—H29B	0.9600
C13—H13C	0.9600	C29—H29C	0.9600
C14—H14A	0.9600	C30—H30A	0.9600
C14—H14B	0.9600	C30—H30B	0.9600
C14—H14C	0.9600	C30—H30C	0.9600
C15—H15A	0.9600	C31—H31A	0.9600
C15—H15B	0.9600	C31—H31B	0.9600
C15—H15C	0.9600	C31—H31C	0.9600
C16—H16A	0.9600	C32—H32A	0.9600
C16—H16B	0.9600	C32—H32B	0.9600
C16—H16C	0.9600	C32—H32C	0.9600
			
O2—C1—C2	59.4 (3)	O1—C17—C18	60.7 (3)
O2—C1—C12	116.3 (4)	O1—C17—C28	116.2 (3)
C2—C1—C12	120.7 (4)	C18—C17—C28	120.6 (4)
O2—C1—C7	120.5 (4)	O1—C17—C23	120.1 (4)
C2—C1—C7	123.8 (4)	C18—C17—C23	124.5 (4)
C12—C1—C7	108.6 (4)	C28—C17—C23	107.8 (4)
C1—O2—C2	61.3 (3)	C17—C18—O1	59.4 (3)
O2—C2—C3	116.8 (4)	C17—C18—C19	123.6 (4)
O2—C2—C1	59.4 (3)	O1—C18—C19	114.6 (4)
C3—C2—C1	123.0 (4)	C17—C18—C29	120.6 (5)
O2—C2—C13	114.9 (4)	O1—C18—C29	114.0 (5)
C3—C2—C13	111.4 (4)	C19—C18—C29	112.5 (5)
C1—C2—C13	120.9 (5)	C18—C19—C20	118.9 (4)
C2—C3—C4	118.7 (4)	C18—C19—H19A	107.6
C2—C3—H3A	107.6	C20—C19—H19A	107.6
C4—C3—H3A	107.6	C18—C19—H19B	107.6
C2—C3—H3B	107.6	C20—C19—H19B	107.6
C4—C3—H3B	107.6	H19A—C19—H19B	107.0
H3A—C3—H3B	107.1	C21—C20—C19	116.6 (4)
C5—C4—C3	116.4 (4)	C21—C20—H20A	108.1
C5—C4—H4A	108.2	C19—C20—H20A	108.1
C3—C4—H4A	108.2	C21—C20—H20B	108.1
C5—C4—H4B	108.2	C19—C20—H20B	108.1
C3—C4—H4B	108.2	H20A—C20—H20B	107.3
H4A—C4—H4B	107.4	C20—C21—C22	118.5 (4)
C4—C5—C6	118.2 (4)	C20—C21—H21A	107.7
C4—C5—H5A	107.8	C22—C21—H21A	107.7
C6—C5—H5A	107.8	C20—C21—H21B	107.7
C4—C5—H5B	107.8	C22—C21—H21B	107.7
C6—C5—H5B	107.8	H21A—C21—H21B	107.1
H5A—C5—H5B	107.1	C21—C22—C31	108.6 (4)
C5—C6—C14	110.0 (4)	C21—C22—C23	114.8 (4)
C5—C6—C15	107.8 (4)	C31—C22—C23	107.5 (4)
C14—C6—C15	107.2 (4)	C21—C22—C30	110.1 (4)
C5—C6—C7	115.3 (4)	C31—C22—C30	107.7 (4)
C14—C6—C7	109.2 (4)	C23—C22—C30	107.9 (4)
C15—C6—C7	106.9 (4)	C17—C23—C24	104.2 (4)
C1—C7—C8	104.2 (4)	C17—C23—C22	122.6 (4)
C1—C7—C6	122.6 (4)	C24—C23—C22	113.3 (3)
C8—C7—C6	113.7 (3)	C17—C23—H23	105.1
C1—C7—H7	104.9	C24—C23—H23	105.1
C8—C7—H7	104.9	C22—C23—H23	105.1
C6—C7—H7	104.9	C25—C24—C23	124.0 (4)
C9—C8—C10	60.1 (3)	C25—C24—C26	59.2 (4)
C9—C8—C7	123.5 (4)	C23—C24—C26	119.7 (4)
C10—C8—C7	120.0 (4)	C25—C24—H24	114.3
C9—C8—H8	114.2	C23—C24—H24	114.3
C10—C8—H8	114.2	C26—C24—H24	114.3
C7—C8—H8	114.2	C24—C25—C26	62.7 (3)
C8—C9—C10	60.8 (3)	C24—C25—Br4	122.1 (4)
C8—C9—Br1	121.6 (3)	C26—C25—Br4	120.0 (4)
C10—C9—Br1	120.2 (3)	C24—C25—Br3	117.4 (3)
C8—C9—Br2	118.2 (3)	C26—C25—Br3	119.6 (4)
C10—C9—Br2	119.7 (3)	Br4—C25—Br3	108.8 (3)
Br1—C9—Br2	109.3 (2)	C25—C26—C32	119.8 (5)
C16—C10—C9	119.5 (4)	C25—C26—C24	58.1 (4)
C16—C10—C8	118.6 (4)	C32—C26—C24	117.6 (4)
C9—C10—C8	59.1 (3)	C25—C26—C27	120.2 (5)
C16—C10—C11	112.4 (4)	C32—C26—C27	113.1 (5)
C9—C10—C11	118.7 (4)	C24—C26—C27	117.4 (4)
C8—C10—C11	118.8 (4)	C28—C27—C26	116.1 (4)
C10—C11—C12	113.7 (4)	C28—C27—H27A	108.3
C10—C11—H11A	108.8	C26—C27—H27A	108.3
C12—C11—H11A	108.8	C28—C27—H27B	108.3
C10—C11—H11B	108.8	C26—C27—H27B	108.3
C12—C11—H11B	108.8	H27A—C27—H27B	107.4
H11A—C11—H11B	107.7	C17—C28—C27	109.9 (4)
C1—C12—C11	110.1 (4)	C17—C28—H28A	109.7
C1—C12—H12A	109.6	C27—C28—H28A	109.7
C11—C12—H12A	109.6	C17—C28—H28B	109.7
C1—C12—H12B	109.6	C27—C28—H28B	109.7
C11—C12—H12B	109.6	H28A—C28—H28B	108.2
H12A—C12—H12B	108.2	C18—C29—H29A	109.5
C2—C13—H13A	109.5	C18—C29—H29B	109.5
C2—C13—H13B	109.5	H29A—C29—H29B	109.5
H13A—C13—H13B	109.5	C18—C29—H29C	109.5
C2—C13—H13C	109.5	H29A—C29—H29C	109.5
H13A—C13—H13C	109.5	H29B—C29—H29C	109.5
H13B—C13—H13C	109.5	C22—C30—H30A	109.5
C6—C14—H14A	109.5	C22—C30—H30B	109.5
C6—C14—H14B	109.5	H30A—C30—H30B	109.5
H14A—C14—H14B	109.5	C22—C30—H30C	109.5
C6—C14—H14C	109.5	H30A—C30—H30C	109.5
H14A—C14—H14C	109.5	H30B—C30—H30C	109.5
H14B—C14—H14C	109.5	C22—C31—H31A	109.5
C6—C15—H15A	109.5	C22—C31—H31B	109.5
C6—C15—H15B	109.5	H31A—C31—H31B	109.5
H15A—C15—H15B	109.5	C22—C31—H31C	109.5
C6—C15—H15C	109.5	H31A—C31—H31C	109.5
H15A—C15—H15C	109.5	H31B—C31—H31C	109.5
H15B—C15—H15C	109.5	C26—C32—H32A	109.5
C10—C16—H16A	109.5	C26—C32—H32B	109.5
C10—C16—H16B	109.5	H32A—C32—H32B	109.5
H16A—C16—H16B	109.5	C26—C32—H32C	109.5
C10—C16—H16C	109.5	H32A—C32—H32C	109.5
H16A—C16—H16C	109.5	H32B—C32—H32C	109.5
H16B—C16—H16C	109.5	C17—O1—C18	60.0 (3)
